# A criterion for strange metallicity in the Lorenz ratio

**DOI:** 10.1038/s41535-023-00598-z

**Published:** 2023-11-07

**Authors:** Evyatar Tulipman, Erez Berg

**Affiliations:** Department of Condensed Matter Physics, Weizmann Institute of Science, 76100 Rehovot, Israel

**Keywords:** Electronic properties and materials, Phase transitions and critical phenomena, Theory and computation

## Abstract

The Wiedemann-Franz (WF) law, stating that the Lorenz ratio *L* = *κ*/(*Tσ*) between the thermal and electrical conductivities in a metal approaches a universal constant ${L}_{0}={\pi }^{2}{k}_{B}^{2}/(3{e}^{2})$ at low temperatures, is often interpreted as a signature of fermionic Landau quasi-particles. In contrast, we show that various models of weakly disordered non-Fermi liquids also obey the WF law at *T* → 0. Instead, we propose using the leading low-temperature correction to the WF law, *L*(*T*) − *L*_0_ (proportional to the inelastic scattering rate), to distinguish different types of strange metals. As an example, we demonstrate that in a solvable model of a marginal Fermi-liquid, *L*(*T*) − *L*_0_ ∝ − *T*. Using the quantum Boltzmann equation (QBE) approach, we find analogous behavior in a class of marginal- and non-Fermi liquids with a weakly momentum-dependent inelastic scattering. In contrast, in a Fermi-liquid, *L*(*T*) − *L*_0_ is proportional to − *T*^2^. This holds even when the resistivity grows linearly with *T*, due to *T* − linear quasi-elastic scattering (as in the case of electron-phonon scattering at temperatures above the Debye frequency). Finally, by exploiting the QBE approach, we demonstrate that the transverse Lorenz ratio, *L*_*xy*_ = *κ*_*xy*_/(*Tσ*_*xy*_), exhibits the same behavior.

## Introduction

The properties of the anomalous normal state of high-*T*_*c*_ superconductors and other quantum materials, commonly dubbed ‘strange metals,’ are one of the most elusive mysteries in condensed matter physics^1,2^. In particular, despite myriad works, it is still unclear to what extent the underlying physics of such systems departs from Landau’s Fermi-liquid (FL) paradigm and necessitates a non-FL (NFL) description.

One of the hallmark characteristics of strange metals is the *T*–linear resistivity at extremely low temperatures. This behavior has been empirically linked with the notion of Planckian dissipation^2–6^, showing a degree of universality throughout different experimental setups and hinting towards a strongly correlated NFL nature for these systems. Albeit at odds with standard FL theory, *T*–linear resistivity can appear in FLs in the presence of certain scattering mechanisms, at least in some intermediate- to low-*T* window^7–10^. It is thus crucial to develop ways to identify the mechanism of *T*–linear resistivity in strange metals.

Here, we present a simple criterion for weakly disordered metals that sharply distinguishes different sources of *T*–linear resistivity. Our criterion is based on the behavior of the low-*T* leading correction to the Lorenz ratio, $L(T)=\frac{\kappa }{T\sigma }$, with *κ* and *σ* being the thermal and electrical conductivities, respectively.

The Weidemann-Franz (WF) law^11^ states that

1 $$\overline{L}\left(T\right)\equiv \frac{L}{{L}_{0}}\to 1$$

as *T* → 0. Here, ${L}_{0}={\pi }^{2}{k}_{B}^{2}/(3{e}^{2})$ is the so-called Lorenz number (we set *e* = *k*_*B*_ = 1 henceforth). Roughly speaking, the deviation of $\overline{L}(T)$ from 1 serves as a measure for the relative contribution of inelastic scattering to charge and thermal transport ($\overline{L}(T)\approx 1$ implies that elastic or quasi-elastic scattering is dominant)^12^. Dominantly inelastic scattering leads to deviations from the WF law in many circumstances^13–16^.

**Fig. 1:**
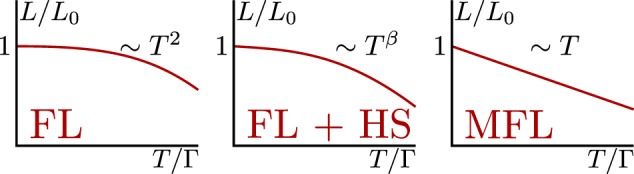
Schematic plot of the low-*T* behavior of the normalized Lorenz ratio for systems that obey the Wiedemann-Franz law at *T* = 0. Here, *T* is assumed to be smaller than Γ, the elastic scattering rate. The *T* dependence of the leading deviation from *L* = *L*_0_ serves as a criterion for strange metallicity: Fermi liquids (FL) exhibit *L*/*L*_0_ − 1 ∝ − *T*^2^; Fermi liquids with hot spots (FL+HS) are characterized by *L*/*L*_0_ − 1 ∝ − *T*^*β*^, 1 < *β* < 2; and certain marginal Fermi-liquids (MFL) have *L*/*L*_0_ − 1 ∝ − *T*.

The validity of the WF law is often used as a test for the existence of FL-like quasi-particle excitations at the lowest temperatures^17–21^. However, the fact that WF is obeyed does not necessarily imply that transport is carried by FL quasi-particles^22–24^. Indeed, as we shall show, one can construct solvable models of NFLs where the WF law is obeyed at *T* → 0. The known mechanisms for *T*–linear resistivity (not necessarily extending down to *T* → 0) in FLs involve elastic or quasi-elastic scattering. These include electron-phonon scattering^9^ or static charged impurities in 2D^7,8^. In contrast, *T*–linear resistivity associated with NFLs is typically associated with inelastic scattering^25–29^. In both cases, however, assuming that (*T*-independent) impurity scattering dominates at sufficiently low *T*, we expect the WF law to be obeyed at *T* → 0. Hence, in order to learn about the FL or NFL origin of the *T*–linear resistivity, one must consider the leading low-temperature deviation from the WF law (see Fig. 1).

Our criterion is applicable to systems that obey the WF law at *T* → 0, as in the cuprates at sufficiently low temperature^17,20,21^. In this context, it is worth noting that certain weakly disordered 2D systems with Coulomb interactions are expected to violate the WF law at *T* → 0^30–32^. However, in metals, the deviation from the WF law is significant at an exponentially small temperature in *k*_*F*_*ℓ*, where *k*_*F*_ is the Fermi momentum and *ℓ* is the elastic mean free path. Our discussion applies above this energy scale.

## Results

### A criterion for strangeness

Consider weakly disordered metals (in 2 or 3 spatial dimensions), such that the dc resistivity has the following form as *T* → 0: $\rho \left(T\right)={\rho }_{0}+A{T}^{\alpha }$, where *ρ*_0_ is the residual resistivity, and *A*, *α* > 0. We assume that impurity scattering dominates at sufficiently low *T*, and the WF law is satisfied at *T* → 0. In this case, the low-*T* electronic thermal resistivity takes the form *ρ*_th_(*T*) ≡ *T*/*κ*_el_ = *ρ*_th,0_ + *BT*^*β*^ with *B*, *β* > 0. The normalized Lorenz ratio (1) then takes the following form:

2 $$\overline{L}\left(T\right)-1\propto -{T}^{\beta }.$$

We claim that the exponent *β* is universal and provides information on the nature of the system. In ordinary FLs, *β* = 2 (logarithmic corrections may arise due to electron-electron interactions in 2D^33,34^). Systems where a portion of Fermi surface (FS) is ‘hot’, while the rest is FL-like, have 1 < *β* < 2. Most interestingly, if *β* ≤ 1, the system is not described by any existing theory of a FL. In particular, the case *α* = *β* = 1 arises in certain models that realize marginal Fermi-liquids (MFLs)^35^. We, therefore, argue that *α* = *β* = 1 could serve as a criterion for ‘strangeness’, in the sense that it signals a full departure from FL theory. See Fig. 1 for a schematic illustration of the different cases.

### Fermi liquids

We consider a weakly disordered FL with electron-electron (el-el) or electron-phonon (el-ph) interactions. We assume that the WF law is obeyed at *T* → 0 due to the dominance of elastic scattering ^12–14,36–39^. Here, and in the following section, the disorder corresponds to static impurities, which provide a source of elastic scattering with rate Γ.

At *T* > 0, el-el and el-ph interactions provide inelastic scattering mechanisms that lead to deviations from the WF law. The contribution of el-el interactions, a hallmark of FL theory, lead to resistivities of the form *ρ* = *ρ*_0_ + *AT*^2^ (assuming Umklapp scattering is present) and *ρ*_th_ = *T*/*κ* = *ρ*_th,0_ + *BT*^2^ (see e.g., refs. ^36,40–42^), which translates to

3 $$\overline{L}\left(T\right)-1\propto -{T}^{2}$$

where the negative slope is related to the additional contribution of forward scattering that relaxes the thermal current, but not the electrical current^12–14^. The el-ph contribution to the electrical (thermal) resistivity is ${{{\mathcal{O}}}}\left({T}^{d+2}\right)$ (${{{\mathcal{O}}}}\left({T}^{d}\right)$), respectively (where *d* > 1), as long as *T* is much smaller than *T*_BG_, the Bloch-Gruneisen temperature^12^. That is, the el-ph contribution is subleading in 3D, while in 2D it may modify the non-universal slope, such that the form (3) holds at sufficiently low *T* in a FL.

In fact, Eq. (3) applies even in cases where the resistivity of a FL is *T*–linear. For example, Coulomb screening of charged impurities, treated within the random phase approximation, leads to a *T*–linear resistivity in a 2D FL, due to thermal suppression of the FL polarizability^8,43^. (In 3D, this contribution to the resistivity is ${{{\mathcal{O}}}}\left({T}^{2}\right)$^7,8,32^). However, in this case, the *T* − linear scattering is still essentially elastic, and the deviations from the WF law still obey Eq. (3).

Unlike the case of charged impurities, *T*–linear resistivity from el-ph interactions emerges only at temperatures *T* ≳ *T*_BG_^12^. Hence, this mechanism is always irrelevant at the limit *T* → 0. On a more practical note, if *T*_BG_ sets a particularly small energy scale, the *T*–linear resistivity due to el-ph scattering might appear to extend down to the lowest experimentally accessible temperatures (as long as *T* ≳ *T*_BG_). However, in this “equipartition” regime, phonons are essentially classical and the el-ph scattering is quasi-elastic. Hence, the WF law is essentially obeyed in this regime^12^.

### Fermi surfaces with hot spots

We now consider systems where a portion of the Fermi surface becomes ‘hot’, i.e., it experiences enhanced scattering with an anomalous *T*–scaling. In some situations, such ‘hot spots’ can lead to an anomalous *T* dependence of the transport coefficients. This situation arises either when the system is on the verge of a finite wavevector instability^44–49^, or when the system is turned to a Van Hove singularity where the topology of the Fermi surface changes^10,15^.

Consider the low–*T* behavior of $\overline{L}\left(T\right)$ in a 2D system where a Van Hove singularity (VHS) crosses the FS in the vicinity of a Lifshitz transition^10,15^. In this case, we refer to the Fermi surface regions near the VHS as ‘hot’. The transport scattering rates are dominated by processes where a ‘cold’ electron (away from the VHS) is scattered by a ‘hot’ one, or two cold electrons are scattered and one of them ends up near the VHS. In clean systems, this leads to $\rho \sim {T}^{2}\log (1/T)$^10,45^ and *ρ*_th_ ~ *T*^3/2^^15^. This behavior persists in the presence of impurities, namely, $\rho ={\rho }_{0}+A{T}^{2}\log (1/T)$ and *ρ*_th_ = *ρ*_th,0_ + *BT*^3/2^^15,45^, such that the deviation from WF law satisfy

4 $$\overline{L}\left(T\right)-1\propto -{T}^{3/2}.$$

We proceed by considering a weakly disordered FL near an antiferromagnetic (AFM) quantum-critical point in 3D, as studied in refs. ^46,47^. In this case, the FS contains ‘hot lines’ connected by the non-zero AFM wavevector, where the scattering off spin fluctuation is most effective. The hot lines then acquire anomalous, NFL-like, scattering rates which may manifest in transport coefficients. In the absence of impurities, these hot lines are short-circuited by the remaining ‘cold’ parts of the FS such that transport coefficients follow the conventional FL behavior at sufficiently low *T*^44^. However, introducing impurities enables the hot lines to participate in transport, since, loosely speaking, the scattering rate is averaged over the entire FS. Ref. ^46^ showed that this leads to an anomalous *T*–scaling of the resistivity, where *ρ* = *ρ*_0_ + *AT*^3/2^ at the lowest temperatures. By extending the analysis of^46^ to the thermal conductivity, we find that the thermal resistivity follows the same anomalous behavior: *ρ*_th_ = *ρ*_th,0_ + *BT*^3/2^, see Supplementary Material. Combining the two resistivities, the deviation from WF law follows Eq. (4).

Interestingly, a straightforward generalization of the analysis above to 2D yields *ρ* = *ρ*_0_ + *AT*^47^. The same reasoning is expected to hold for the thermal resistivity, which would imply that $\overline{L}-1\propto -T$ in 2D. However, this analysis is based on the Hertz-Millis treatment of the AFM QCP, which breaks down at sufficiently low temperatures in the 2D case^50,51^.

### Marginal Fermi liquids

In this section, we construct a solvable model of a 2D weakly disordered MFL that shows *T*–linear resistivity down to the lowest temperatures and obeys the WF law at *T* → 0, with a leading correction of the form

5 $$\overline{L}\left(T\right)-1\propto -T.$$

In addition, we comment on the expected behavior of other tractable models of MFLs in 2 and 3 dimensions, suggesting that Eq. (5) could be a robust signature of a class of weakly disordered MFLs. We further corroborate this expectation using the Quantum Boltzmann Equation (QBE) approach in the following section.

Consider a weakly disordered variant of the model studied in ref. ^28^, based on a 2-band lattice generalization of the Sachdev-Ye-Kitaev (SYK) model^52–54^. The model is defined on a D–dimensional lattice, and contains two species of fermions, {*c*} and {*f*}, each containing *N* orbitals per unit cell, governed by the Hamiltonian *H* = *H*_*c*_ + *H*_*f*_ + *H*_*cf*_, where

6 $$\begin{array}{l}{H}_{c}\,=\,-\mathop{\sum}\limits_{{{{\boldsymbol{r}}}},{{{{\boldsymbol{r}}}}}^{{\prime} },l}\left({t}_{{{{\boldsymbol{r}}}},{{{{\boldsymbol{r}}}}}^{{\prime} }}+\mu {\delta }_{{{{\boldsymbol{r}}}},{{{{\boldsymbol{r}}}}}^{{\prime} }}\right){c}_{{{{\boldsymbol{r}}}}l}^{{\dagger} }{c}_{{{{{\boldsymbol{r}}}}}^{{\prime} }l}+\frac{1}{{N}^{1/2}}\mathop{\sum}\limits_{{{{\boldsymbol{r}}}},ij}{W}_{ij{{{\boldsymbol{r}}}}}{c}_{{{{\boldsymbol{r}}}}i}^{{\dagger} }{c}_{{{{\boldsymbol{r}}}}j};\\ {H}_{cf}\,=\,\frac{1}{{N}^{3/2}}\mathop{\sum}\limits_{{{{{\boldsymbol{r,r}}}}}^{{\prime} }}\mathop{\sum}\limits_{ijkl}{V}_{ijkl}{\Upsilon }_{{{{{\boldsymbol{r,r}}}}}^{{\prime} }}{c}_{{{{\boldsymbol{r}}}}i}^{{\dagger} }{f}_{{{{{\boldsymbol{r}}}}}^{{\prime} }j}^{{\dagger} }{c}_{{{{\boldsymbol{r}}}}k}{f}_{{{{{\boldsymbol{r}}}}}^{{\prime} }l};\\ {H}_{f}\,=\,\frac{2}{{N}^{3/2}}\mathop{\sum}\limits_{ijkl}{U}_{ijkl}{f}_{{{{\boldsymbol{r}}}}i}^{{\dagger} }{f}_{{{{\boldsymbol{r}}}}j}^{{\dagger} }{f}_{{{{\boldsymbol{r}}}}k}{f}_{{{{\boldsymbol{r}}}}l}.\end{array}$$

The hopping matrix ${t}_{{{{\boldsymbol{r}}}},{{{{\boldsymbol{r}}}}}^{{\prime} }}$ is diagonal in orbital space and depends only on the distance $\left\vert {{{\boldsymbol{r}}}}-{{{{\boldsymbol{r}}}}}^{{\prime} }\right\vert$. The last term in *H*_*c*_ describes on-site disorder for the *c*-fermions, where *W*_*ij****r***_ are site-dependent Gaussian random independent potential, satisfying $\overline{{W}_{ij{{{\boldsymbol{r}}}}}}=0,\overline{{W}_{ij{{{\boldsymbol{r}}}}}{W}_{ij{{{{\boldsymbol{r}}}}}^{{\prime} }}}={W}^{2}{\delta }_{{{{\boldsymbol{r}}}},{{{{\boldsymbol{r}}}}}^{{\prime} }}$. The couplings in *H*_*cf*_ and *H*_*f*_ are site-independent Gaussian random independent variables, satisfying $\overline{{V}_{ijkl}}=0,\overline{{V}_{ijkl}^{2}}={U}_{cf}^{2}$ and similarly for *U*_*ijkl*_ (with variance ${U}_{f}^{2}$). The function ϒ determines the spatial dependence of the *cf*-interaction. Note that for *W* = 0, the model is translationally invariant for every realization of the interactions. We first consider the case of on-site interaction as in^28^: ${\Upsilon }_{{{{{\boldsymbol{r,r}}}}}^{{\prime} }}={\delta }_{{{{{\boldsymbol{r,r}}}}}^{{\prime} }}$. Spatially extended ϒ will be considered later on.

The model is solvable in the *N* → *∞* limit, where its properties are dictated by replica-diagonal saddle-point of the real- and imaginary-time effective action^28^. The low-energy saddle-point equations describe SYK-like, incoherent *f*-fermions. These *f*-fermions constitute a local quantum-critical bath for the *c*-fermions, giving rise to a weakly disordered MFL form for the Green’s function of the *c*-fermions. Importantly, the on-site disorder *W* for the *c*-fermions does not alter the low-energy behavior of the *f*-fermions, rather it only enters as an additional *T*-independent, elastic scattering term to the *c*-fermions. For example, at *T* = 0, the Matsubara frequency Green’s function is of the form

7 $${G}_{c}\left({{{\boldsymbol{k}}}},i\omega \right)=\frac{1}{i\omega -{\varepsilon }_{{{{\boldsymbol{k}}}}}-{\Sigma }_{c}\left(i\omega \right)};$$

8 $${\Sigma }_{c}\left(i\omega \right)=-i\frac{\Gamma }{2}\,{{\mbox{sgn}}}\,\left(\omega \right)+{\Sigma }_{cf}\left(i\omega \right);$$

9 $${\Sigma }_{cf}\left(i\omega \right)=-\frac{{\nu }_{0}{U}_{cf}^{2}}{2{\pi }^{2}{U}_{f}}i\omega \log \left(\frac{{U}_{f}}{\left\vert \omega \right\vert }\right),$$

where Γ = 2*πν*_0_*W*^2^ is the disorder energy scale.

We proceed to consider transport. We compute the electrical and thermal conductivities using the Kubo formula. By virtue of the locality of the *f*-fermions, both conductivities are given in terms of the bare bubble expressions, similarly to refs. ^28,29^. We obtain the thermal conductivity,

10 $$\kappa =\frac{{v}_{F}^{2}{\nu }_{0}}{16{T}^{2}}\int\frac{d\epsilon }{2\pi }\frac{{\epsilon }^{2}}{\left\vert {\Sigma }_{R}^{{\prime\prime} }\left(\epsilon \right)\right\vert }{{{\mbox{sech}}}}^{2}\left(\frac{\epsilon }{2T}\right)$$

and the electrical conductivity,

11 $$\sigma =\frac{{v}_{F}^{2}{\nu }_{0}}{16T}\int\frac{d\epsilon }{2\pi }\frac{1}{\left\vert {\Sigma }_{R}^{{\prime\prime} }\left(\epsilon \right)\right\vert }{{{\mbox{sech}}}}^{2}\left(\frac{\epsilon }{2T}\right).$$

**Fig. 2:**
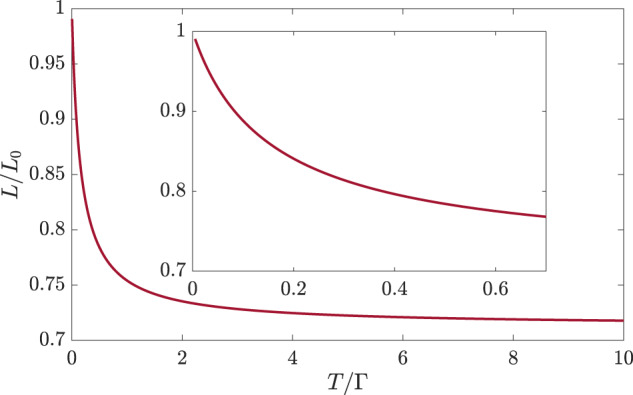
The Lorenz ratio as a function of temperature for the MFL model (6) with local *cf*-interaction. Γ is the elastic scattering rate. The inset shows the limit *T* ≪ Γ which obeys Eq. (5).

The imaginary part of the retarded self-energy is given by $-{\Sigma }_{R}^{{\prime\prime} }\left(\epsilon \right)=\frac{\Gamma }{2}+{g}^{2}{{{\rm{Im}}}}\left[\epsilon \psi \left(\frac{-i\epsilon }{2\pi T}\right)+i\pi T\right]$ with ${g}^{2}=\frac{{U}_{cf}^{2}{\nu }_{0}}{2{\pi }^{2}{U}_{f}}$ and where *ψ*(*z*) is the digamma function^29^. Using Eq. (10) and Eq. (11), we find that the WF law is obeyed at *T* → 0, despite the fact that the MFL description of the *c*-fermions persists to the lowest temperatures, and that the leading deviation from the WF law obeys Eq. (5). The Lorenz ratio *L*(*T*)/*L*_0_ as a function of *T* is shown in Fig. 2. As can be seen in the figure, *L*/*L*_0_ decreases linearly with *T* at small *T*, and saturates to the value corresponding to the clean case, *L*/*L*_0_ ≈ 0.71^29,55^, at *T* ≳ Γ.

In order to examine the robustness of these results to details of the model, we consider the addition of spatially extended *cf*-interactions: ${\Upsilon }_{{{{\boldsymbol{r}}}},{{{{\boldsymbol{r}}}}}^{{\prime} }}={\delta }_{{{{\boldsymbol{r}}}},{{{{\boldsymbol{r}}}}}^{{\prime} }}+\eta {\sum }_{{{{\boldsymbol{\delta }}}} = \pm \hat{x},\pm \hat{y}}{\delta }_{{{{\boldsymbol{r}}}},{{{{\boldsymbol{r}}}}}^{{\prime} }+{{{\boldsymbol{\delta }}}}}$ with *η* being a small control parameter. This modification does not change the MFL form of the self-energy of the *c*-fermions. In addition, the form of the thermal current operator is unchanged, see Supplementary Material. Hence, to leading order in *η*, the conductivities are given by *α*_*η*_ = *α*_0_ + *δα* for *α* = *σ*, *κ*, where we have denoted *α*_*η*=0_ ≡ *α*_0_, and the correction *δα* is ${{{\mathcal{O}}}}\left(\eta \right)$ and corresponds to the current bubble with an insertion of a single *cf*-interaction rung, see Supplementary Material. These corrections alter the Lorenz ratio, such that for *T* ≫ Γ,

12 $$L=\frac{{\kappa }_{0}}{T{\sigma }_{0}}\left(1+\frac{\delta \kappa }{{\kappa }_{0}}-\frac{\delta \sigma }{{\sigma }_{0}}\right)\ne \frac{{\kappa }_{0}}{T{\sigma }_{0}},$$

which demonstrates that the saturation value is not universal. Importantly, the spatially extended *cf*-interaction does not alter the *T* → 0 behavior of the Lorenz ratio, which obeys Eq. (5). We will demonstrate this and further highlight the conditions for which Eq. (5) is valid within the framework of the QBE in the next section.

It is worth commenting that the simplicity of the analysis of (6) comes with a price in the form of a residual *T* → 0 extensive entropy due to the SYK-nature of the *f*-fermions^28,29,53,54^. The residual entropy is relieved upon allowing quadratic terms in the *f*-fermions, but these also lead to FL behavior at low temperatures^28^. Nevertheless, we expect Eq. (5) to be a robust property of weakly disordered MFLs in 2 and 3 dimensions that show *T*–linear electrical resistivity, as we discuss in the next section.

Let us briefly note that the results presented here and in the next section can be generalized to *f*-fermions governed by an SYK_*q*_ (*q* > 4) Hamiltonian, while the *cf*-interaction is unchanged. For *q* > 4, the *c*-fermions realize an incoherent, NFL description with *ρ* = *ρ*_0_ + *AT*^4/*q*^ and *ρ*_th_ = *ρ*_th,0_ + *BT*^4/*q*^, such that $\overline{L}-1\propto -{T}^{4/q}$^28^.

### Quantum Boltzmann equation approach

Even in the absence of well-defined quasi-particles, we may still derive a QBE for a generalized Fermi distribution function in the model of the previous section. Here we briefly outline the idea behind the QBE approach for MFLs and the conditions for which it is applicable. In addition, we highlight its implications on the validity of the WF law and the criterion for strangeness in a certain class of MFLs, using a generalization of the model (6) as a simple representative. We elaborate on several issues and supply technical details in the Supplementary material.

To derive a QBE in the absence of well-defined quasi-particles, we utilize the MFL form of the self-energy and the fact that the spectral function of the *c*-fermions is sharply peaked at the FS as a function of *ε*_***k***_ (this is in contrast to the QBE approach for FLs which relies on the sharp quasi-particle peak as a function of *ω*). Within this approximation, known as the Prange-Kadanoff (PK) reduction scheme^56,57^, the momenta of the *c*-fermions are restricted to the FS. Roughly speaking, the PK reduction is valid when the width of the electronic spectral function ${{{\mathcal{A}}}}(\omega \sim T,{{{\boldsymbol{k}}}})$ as a function of ***k*** is smaller than the typical momentum transfer in both elastic and inelastic scattering events, see Supplementary Material and ref. ^58^.

Considering the MFL model (6), the QBE approach illustrates that

(i) The WF law may hold at *T* → 0 due to the dominance of elastic scattering, regardless of the existence of well-defined quasi-particles;
(ii) The leading deviation from the WF law obeys Eq. (5) in weakly disordered MFLs that admit the PK reduction scheme;

where (ii) can be understood as a consequence of Matthiessen’s rule. We further find that the deviation in Eq. (5) holds for a class of generalized models with spatially extended *cf*-interactions, see e.g., the previous section, which confirms that (i) and (ii) have a much broader regime of validity in weakly disordered MFLs (and NFLs). Specifically, assuming that the momentum-dependence of the inelastic scattering rate is sufficiently weak (as defined above), such that PK reduction can be applied, the QBE approach suggests that WF law should hold at *T* → 0. Moreover, since in these circumstances, the transport relaxation rate is proportional to the single particle scattering rate, the leading low-*T* deviation from the WF law is expected to satisfy Eq. (5).

### Transverse Lorenz ratio

We employ the QBE approach to generalize our discussion to the transverse Lorenz ratio:

13 $${L}_{xy}\equiv \frac{{\kappa }_{xy}}{T{\sigma }_{xy}},$$

where *σ*_*xy*_ and *κ*_*xy*_ denote the transverse electrical and thermal conductivities, respectively. Specifically, by solving the linearized QBE of the weakly disordered MFLs (6), we find that the leading deviation from the (transverse) WF law for a class of MFLs follows the same scaling as the longitudinal:

14 $${\overline{L}}_{xy}-1\propto -T,$$

as in Eq. (5); see Supplementary Material. Moreover, while the derivation of the transverse conductivities is slightly more involved due to the presence of a weak magnetic field, the key ingredient remains the validity of the PK reduction scheme. This has the remarkable implication that, as long as the PK reduction scheme is valid, our conclusions for the longitudinal Lorenz ratio (i.e., (i) and (ii) from the previous section) equally apply to the transverse Lorenz ratio of weakly disordered MFLs (or NFLs). In addition, while the transverse conductivities are proportional to the applied magnetic field, this proportionality factor cancels in *L*_*xy*_ such that the leading deviation is independent of the magnetic field.

Note further that the extension of our criterion to the transverse Lorenz ratio holds also for weakly disordered FLs, where the leading deviation satisfies *L*_*xy*_ − *L*_0_ ∝ − *T*^2^^12^. The same conclusion is expected to hold for Fermi surface with hot spots since, within the conventional Boltzmann transport theory (for sufficiently weak magnetic field that can be treated perturbatively), the dominant inelastic scattering rate that governs longitudinal transport also governs transverse transport.

## Discussion

Naively, one may have expected the WF law to hold at *T* → 0 only in weakly disordered Fermi liquids with well-defined quasi-particles. This is because, within the conventional Landau-Boltzmann description of transport, the universal value *L*_0_ originates from integrating over Fermi functions, implying that the existence of well-defined quasi-particles is necessary. In contrast, as shown in this work, a broad class of weakly disordered non-Fermi-liquid metals with no well-defined quasi-particles (in the sense that the electron scattering rate is either comparable to or larger than, the energy) also satisfy the WF law at *T* → 0. Intuitively, the fact that this class of systems obey the Wiedemann-Franz law may be understood from the fact that, while there is no well-defined Fermi surface with a sharp jump in the fermion momentum occupation function, the generalized energy distribution function $f\left(\omega \right)=-i\int\frac{d\varepsilon }{2\pi }{G}^{ < }\left(\varepsilon ,\omega \right)$, is a Fermi function (see Supplementary Material). A sufficient condition for the WF law to hold is that the QBE approach is applicable; this requires, in particular, that (i) The width of electronic spectral functions at zero energy is smaller than the Fermi momentum, and that (ii) The dependence of the electronic scattering rate on momentum is non-singular. Note that, in particular, condition (i) implies that the resistivity is small compared to the Mott-Ioffe-Regel limit.

Thus, the fact the WF is obeyed at *T* = 0 is not sufficient to deduce that these systems are conventional Fermi liquids in disguise. Instead, we propose to examine the deviation of the Lorenz ratio *L*(*T*) from *L*_0_ as *T* → 0. Since this quantity depends on the degree of inelastic scattering, it can distinguish different sources of strange metallicity, such as Fermi liquids with a source of *T* − linear nearly elastic scattering (such scattering from an Einstein bosonic mode whose frequency is lower than *T*), from “true” non-Fermi liquids where the scattering is inelastic (see Fig. 1).

In practice, our criterion is applicable under experimental conditions where the electronic degrees of freedom dominate heat transport at low *T*. For the longitudinal case, while these conditions can be met in some scenarios (for example refs. ^42,59^), it could also be the case that other degrees of freedom, e.g., phonons, will dominate the thermal conductivity which would make our criterion inaccessible. To separate the electronic contribution, the transverse Lorenz ratio *L*_*xy*_ is often used (since *κ*_*xy*_ is often, although not always^60^, dominated by the electronic contribution). Here we showed that our criterion applies to the longitudinal and transverse cases at once, and therefore expect it to be widely applicable.

An intriguing issue concerns the application of our criterion to theories of quantum-critical metals, especially in cases where the electrical resistivity is *T* − linear^27,61,62^. In this regard, we point out ref. ^59^, which reported low–*T* transport measurements in a weakly disordered 3D system at a ferrmomagnetic critical point. It was found that at low *T*, *ρ* = *ρ*_0_ + *AT*^5/3^ while *ρ*_th_ = *ρ*_th,0_ + *BT*, such that $\overline{L}-1\propto -T$, consistent with MFL behavior by our criterion^51,63^. This observation is further corroborated by evidence for a $T\log \left(1/T\right)$ behavior in the specific heat^64^, as expected for a MFL^35^.

## Methods

All analytical calculations are explicitly presented in the Supplementary note.

### Supplementary information

SUPPLEMENTAL MATERIAL

## Data Availability

The data that support the findings of this study are available from the authors on request.
