# Supplementary material for: A criterion for strange metallicity in the Lorenz ratio
Source: NPJ Quantum Mater. 2023 Nov 7;8(1):66. doi: 10.1038/s41535-023-00598-z (PMC11041806; doi:10.1038/s41535-023-00598-z)
Supplement: Supplementary file 1 — SUPPLEMENTAL MATERIAL [file 41535_2023_598_MOESM1_ESM.pdf]

Supplementary Material for A Criterion for Strange Metallicity in the Lorenz Ratio

Evyatar Tulipman[1,*] and Erez Berg[1]

[1]*Department of Condensed Matter Physics, Weizmann Institute of Science, Rehovot 76100, Israel*

(Dated: November 6, 2023)

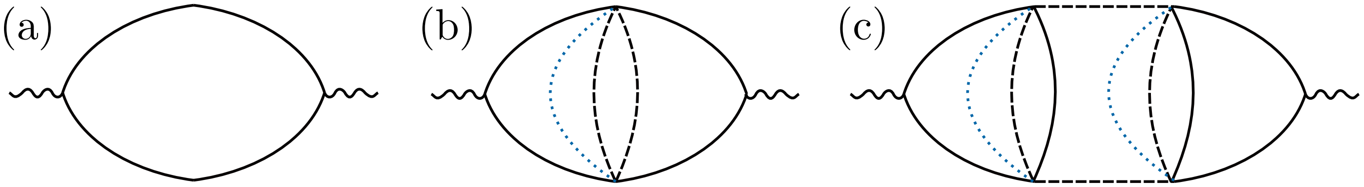

Supplementary Figure 1. Leading diagrams in $1/N$ for the electrical and thermal current-current correlation function. Solid line correspond to c-fermions, Dashed lines correspond to f-fermions and blue dotted lines denote averaging over realizations of V_{ijkl}.

THERMAL CONDUCTIVITY IN A FL NEAR AFM CRITICALITY IN 3D

We consider the model studied in Ref. [1]. In the framework of the variational Boltzmann approach [2], the low-T inverse electronic thermal conductivity is given by

$$\frac{1}{\kappa}=\frac{1}{\kappa_{\text{imp}}}+\frac{\int_{\boldsymbol{k},\boldsymbol{k'}}\left(\Phi_{\boldsymbol{k}}-\Phi_{\boldsymbol{k}'}\right)^{2}W_{\boldsymbol{k}\boldsymbol{k'}}}{\left|\int_{\boldsymbol{k}}\boldsymbol{v}_{\boldsymbol{k}}\left(\varepsilon_{\boldsymbol{k}}-\mu\right)\Phi_{\boldsymbol{k}}\frac{\partial f_{\boldsymbol{k}}^{0}}{\partial\varepsilon_{\boldsymbol{k}}}\right|^{2}}. \tag{1}$$

Here $\frac{1}{\kappa_{\text{imp}}}$ is the contribution from static impurities that, when combined with the residual electrical resistivity, satisfies the WF law at $T\to0$. In addition, $\boldsymbol{v}_{\boldsymbol{k}}=\partial_{\boldsymbol{k}}\varepsilon_{\boldsymbol{k}}$ where $\varepsilon_{\boldsymbol{k}}$ is the dispersion relation, μ is the chemical potential. We are interested in the leading deviation from the WF law, associated with the second term in Eq. (1). We mainly follow the notation of [2], where the full out-of-equilibrium distribution function is given by $f_{\boldsymbol{k}}=f_{\boldsymbol{k}}^{0}+\Phi_{\boldsymbol{k}}\frac{\partial f_{\boldsymbol{k}}^{0}}{\partial\varepsilon_{\boldsymbol{k}}}$. f^{0} (n) denotes the equilibrium Fermi (Bose) distribution function. The transition rate associated with critical AFM spin fluctuations is given by $W_{\boldsymbol{k}\boldsymbol{k}'}=2g_{S}^{2}f_{\boldsymbol{k}}^{0}\left(1-f_{\boldsymbol{k}'}^{0}\right)n\left(\varepsilon_{\boldsymbol{k}'}-\varepsilon_{\boldsymbol{k}}\right)\text{Im}\chi\left(\boldsymbol{k}'-\boldsymbol{k},\varepsilon_{\boldsymbol{k}}-\varepsilon_{\boldsymbol{k}'}\right)$, with the retarded spin correlation function $\chi\left(\boldsymbol{q},\omega\right)=\sum_{\pm}\frac{1}{1/(q_{0}\xi)^{2}+(\boldsymbol{q}\pm\boldsymbol{Q})^{2}/q_{0}^{2}-i\omega/\Gamma}$ [1].

To proceed we use the ansatz $\Phi_{\boldsymbol{k}}=\eta\left(\varepsilon_{\boldsymbol{k}}-\mu\right)\boldsymbol{u}\cdot\boldsymbol{k}$, with a small η and where $\boldsymbol{u}$ is a unit-vector in the direction heat current, which is appropriate at the low-T limit, where impurity scattering dominates κ. From this point the analysis is analogous to the one in Refs. [1, 3], namely, the low-T scaling of $1/\kappa$ is determined by the T-dependent phase space of the hot-lines that scales as $\sqrt{T}$. In total, we obtain that the thermal conductivity is given by

$$\frac{1}{\kappa}\;=\;\frac{1}{\kappa_{\text{imp}}}+\frac{1}{\kappa_{\text{spin}}}=\frac{A}{T}+B\sqrt{T}, \tag{2}$$

where A and B are related to the impurity and spin contributions. Finally the thermal resistivity obeys $\rho_{\text{th}}=T/\kappa=\rho_{\text{th},0}+BT^{3/2}$.

SPATIALLY EXTENDED cf-INTERACTION

We discuss the modifications to the self-energy and resistivities in the presence of a spatially extended cf-interaction.

Self energy

As in the main text, we follow the notation of [4]. We first show that the MFL form of the c-fermions self-energy is unchanged by extending the range of the cf-interactions, up to a non-universal numerical coefficient. Let us set $\Upsilon_{\boldsymbol{r},\boldsymbol{r}'}=\delta_{\boldsymbol{r},\boldsymbol{r}'}+\eta\sum_{\boldsymbol{\delta}=\pm\hat{x},\pm\hat{y}}\delta_{\boldsymbol{r},\boldsymbol{r}'+\boldsymbol{\delta}}$ as in the main text. To show that the self-energy is unchanged, we note that to leading order in η, the cf-contribution to the self-energy of the c-fermions is given by

$$\Sigma_{cf}\left(\boldsymbol{k},i\omega\right)=-U_{cf}^{2}\int_{\boldsymbol{q},\Omega}G_{c}\left(\boldsymbol{k}+\boldsymbol{q},i\omega+i\Omega\right)\widetilde{\Upsilon}\left(\boldsymbol{q}\right)\Pi_{f}\left(i\Omega\right), \tag{3}$$

where $\widetilde{\Upsilon}(\boldsymbol{q}) = 1 + 4\eta(\cos q_x + \cos q_y) + \mathcal{O}(\eta^2)$. Considering $\boldsymbol{k}$ on the FS, we approximate $\varepsilon_{\boldsymbol{k}+\boldsymbol{q}} \approx v_F q \cos\theta$ where θ is the angle between $\boldsymbol{k}$ and $\boldsymbol{q}$. In addition, to obtain the most singular contribution we further approximate $\widetilde{\Upsilon}(\boldsymbol{q}) \approx 1 + 8\eta + \mathcal{O}(q^2)$. Carrying the momentum and frequency integrals in the usual way (see [4] for details), it follows that, in the low-energy limit,

$$\Sigma_{cf}(\boldsymbol{k}, i\omega) = -i(1+\alpha_\Upsilon)\frac{U_{cf}^2\nu_0}{2\pi U_f}\left[\log\left(\frac{U_f}{|\omega|}\right) - 1\right]\omega \tag{4}$$

where $\alpha_\Upsilon = 8\eta$, namely, the MFL form of the self-energy is unchanged.

Thermal current operator

Note that the thermal current is given by $\boldsymbol{j}_{\text{th}}(t) = \frac{i}{2}\sum_l \int_{\boldsymbol{k}} \partial_{\boldsymbol{k}}\varepsilon_{\boldsymbol{k}}\left[\dot{c}^\dagger_{\boldsymbol{k}l}c_{\boldsymbol{k}l} - c^\dagger_{\boldsymbol{k}l}\dot{c}_{\boldsymbol{k}l}\right]$ as long as the cf-interaction is local in the c-fermions. For brevity, let us show this in a simplified 1D continuum model given by

$$H = \frac{1}{2m}\int_r \partial_r c_r^\dagger \partial_r c_r + \int_{r,r'} \widehat{V}_{r,r'} c_r^\dagger c_r, \tag{5}$$

where $\widehat{V}_{r,r'} = V\Upsilon_{r,r'} f^\dagger_{r'} f_{r'}$. We use the continuity equation to identify the thermal current operator: $\dot{h}_r = \partial_r j_{\text{th},r}$. Here the local Hamiltonian density is given by

$$h_r = \partial_r c_r^\dagger \partial_r c_r + \int_{r'} \widehat{V}_{r,r'} c_r^\dagger c_r \tag{6}$$

and $\dot{h}_r = i[h_r, H]$. Ignoring the time derivatives of the f-fermions, which do not participate in transport, we have that

$$\dot{h}_r = \frac{1}{2m}\left(\partial_r \dot{c}_r^\dagger \partial_r c_r + \partial_r c_r^\dagger \partial_r \dot{c}_r\right) + \int_{r'} \widehat{V}_{r,r'}\left(\dot{c}^\dagger_{rk} c_{rl} + c^\dagger_{rk}\dot{c}_{rl}\right). \tag{7}$$

To proceed we rewrite the first term as $\partial_r \dot{c}_r^\dagger \partial_r c_r + \partial_r c_r^\dagger \partial_r \dot{c}_r = \partial_r\left(\dot{c}_r^\dagger \partial_r c_r + \partial_r c_r^\dagger \dot{c}_r\right) - \left(\dot{c}_r^\dagger \partial_r^2 c_r + \partial_r^2 c_r^\dagger \dot{c}_r\right)$. Then, we insert the equation of motion for $c_r, c_r^\dagger$ (i.e. $\partial_r^2 c_r = -i\dot{c}_r - \int_{r'}\widehat{V}_{r,r'}c_r$), which is *local* as long as the cf-interaction is local in the c-fermions, and arrive at

$$\dot{h}_r = \frac{1}{2m}\partial_r\left(\dot{c}_r^\dagger \partial_r c_r + \partial_r c_r^\dagger \dot{c}_r\right) = \partial_r j_{\text{th},r}, \tag{8}$$

with $j_{\text{th},r} = \frac{1}{2m}\left(\dot{c}_r^\dagger \partial_r c_r + \partial_r c_r^\dagger \dot{c}_r\right)$. Going back to the lattice, the total thermal current is indeed given by $j_{\text{th}} = \frac{i}{2}\int_k \partial_k \varepsilon_k \left[\dot{c}_k^\dagger c_k - c_k^\dagger \dot{c}_k\right]$. Generalizing this to higher dimensions is straightforward.

Vertex corrections

Consider the effect of the nearest-neighbors (n.n.) interaction on the conductivities. Here we demonstrate the effect on the electrical conductivity. The thermal conductivity follows exactly the same considerations. First recall that in the absence of n.n. cf-interaction, all vertex corrections vanish due to the locality of the f-fermions. The conductivities are then associated with the current-current bubble diagram shown in Fig. 1a. Adding n.n. interactions lead to an additional contribution, which is associated with the bubble diagram with a single rung inserted, see Fig. 1b. Importantly, while the diagram shown in Fig. 1c is the same order in $1/N$, it does not contribute to the conductivities because it is non-zero only at second order in η.

For our purposes, it is sufficient to show that the contribution of Fig. 1b does not vanish. It then follows that, to leading order in η, there are vertex corrections to σ and κ that generically alter the high-T ($T \gg \Gamma$) saturation value. The contribution due to the insertion of a single rung is given by

$$\delta\Pi_J^x(i\Omega) = U_{cf}^2 \int_{\omega,\omega',\boldsymbol{k},\boldsymbol{k'}} v^x_{\boldsymbol{k}} v^x_{\boldsymbol{k'}} G_c(i\omega + i\Omega, \boldsymbol{k}) G_c(i\omega, \boldsymbol{k}) \widetilde{\Upsilon}(\boldsymbol{k} - \boldsymbol{k'}) \Pi_f(i\omega - i\omega') G_c(i\omega' + i\Omega, \boldsymbol{k'}) G_c(i\omega', \boldsymbol{k'}). \tag{9}$$

We can now see that in the absence of n.n. interaction, i.e., when $\widetilde{\Upsilon}=1$, this contribution vanishes since the integrand is odd in $\boldsymbol{k}$ and in $\boldsymbol{k}'$. However upon introducing the n.n. interaction, there is an additional odd part in $\boldsymbol{k}$ and in $\boldsymbol{k}'$:

$$\widetilde{\Upsilon}\left(\boldsymbol{k}-\boldsymbol{k}'\right)\ =4\eta\sin k_x\sin k_x'+\left(\text{terms even in }k_x,k_x'\right). \tag{10}$$

The product of the odd part of $\widetilde{\Upsilon}$ with the velocities is even in $\boldsymbol{k}$ and $\boldsymbol{k}'$ and therefore does not vanish under the integral. Note that only taking the leading diagrams in η is equivalent to a perturbative treatment of the current vertex, which justified since we are perturbing about a non-singular point, i.e., since the conductivities are finite at $\eta=0$.

High-T saturation value

We recall the Kubo formula for the dc electrical conductivity, expressed in terms of the analytic continuation of the Matsubara frequency current-current correlator:

$$\sigma\left(T\right)=\lim_{\Omega\to0}\lim_{\eta\to0}\frac{\mathrm{Im}\Pi_J^x\left(i\Omega_l\to\Omega+i\eta\right)}{\Omega}. \tag{11}$$

For any $T>0$, we can thus define dimensionless frequencies $\overline{\Omega}_l=\Omega_l/T$ and $\overline{\Omega}=\Omega/T$ such that

$$\sigma\left(T\right)=\lim_{\overline{\Omega}\to0}\lim_{\eta\to0}\frac{\mathrm{Im}\Pi_J^x\left(i\overline{\Omega}_l\to\overline{\Omega}+i\eta\right)}{T\overline{\Omega}}. \tag{12}$$

Therefore, obtaining the T-scaling of $\mathrm{Im}\Pi_J^x\left(i\overline{\Omega}_l\to\overline{\Omega}+i\eta\right)$ yields the T-scaling of σ. We will now show that this simple power counting argument can be used to obtain the T-scaling of the conductivity correction.

To begin, consider the expression for the current correlator related to the conductivity correction:

$$\delta\Pi_J^x\left(i\Omega_l\right)\ =\ U_{cf}^2T^2\sum_{m,n\in\mathbb{Z}}\int_{\boldsymbol{k},\boldsymbol{k}'}v_{\boldsymbol{k}}^xv_{\boldsymbol{k}'}^x \tag{13}$$

$$G_c\left(i\omega_n+i\Omega_l,\boldsymbol{k}\right)G_c\left(i\omega_n,\boldsymbol{k}\right)\widetilde{\Upsilon}\left(\boldsymbol{k}-\boldsymbol{k}'\right)\Pi_f\left(i\omega_n-i\omega_m\right)G_c\left(i\omega_m+i\Omega_l,\boldsymbol{k}'\right)G_c\left(i\omega_m,\boldsymbol{k}'\right) \tag{14}$$

We take the odd part of $\widetilde{\Upsilon}$, as above, and approximate $\sin k_x\sim\frac{1}{W_c}v_{\boldsymbol{k}}^x$:

$$\delta\Pi_J^x\left(i\Omega\right)\ \sim\ \eta\frac{U_{cf}^2}{W_c^2}T^2\sum_{m,n\in\mathbb{Z}}\int_{\boldsymbol{k},\boldsymbol{k}'}\left(v_{\boldsymbol{k}}^x\right)^2\left(v_{\boldsymbol{k}'}^x\right)^2 \tag{15}$$

$$G_c\left(i\omega_n+i\Omega_l,\boldsymbol{k}\right)G_c\left(i\omega_n,\boldsymbol{k}\right)\Pi_f\left(i\omega_n-i\omega_m\right)G_c\left(i\omega_m+i\Omega_l,\boldsymbol{k}'\right)G_c\left(i\omega_m,\boldsymbol{k}'\right). \tag{16}$$

This allows us to change the momentum integration to energy integration in the usual way. Then, by expressing the Green's functions with the spectral representation and integrating over ε,ε', we obtain

$$\delta\Pi_J^x\left(i\Omega_l\right)\ \sim\ \eta\frac{U_{cf}^2}{W_c^2}\nu_0^2v_F^4\int_{\epsilon_1,\epsilon_2,\epsilon_3,\epsilon_3,\epsilon_5}\mathcal{S}_c\left(\epsilon_1,\epsilon_2\right)\mathcal{F}\left(\epsilon_3\right)\mathcal{S}_c\left(\epsilon_4,\epsilon_5\right). \tag{17}$$

$$\times T^2\sum_{n,m\in\mathbb{Z}}\frac{1}{\epsilon_1-i\Omega_l-i\omega_n}\frac{1}{\epsilon_2-i\omega_n}\frac{1}{\epsilon_3-i\omega_n+i\omega_m}\frac{1}{\epsilon_4-i\Omega_l-i\omega_m}\frac{1}{\epsilon_5-i\omega_m}, \tag{18}$$

where

$$\mathcal{S}_c\left(\epsilon_i,\epsilon_j\right)\equiv\frac{\Sigma_R''\left(\epsilon_i\right)+\Sigma_R''\left(\epsilon_j\right)}{\left(\epsilon_i-\epsilon_j+\Sigma_R'\left(\epsilon_i\right)-\Sigma_R'\left(\epsilon_j\right)\right)^2+\left(\Sigma_R''\left(\epsilon_i\right)+\Sigma_R''\left(\epsilon_j\right)\right)^2} \tag{19}$$

and $\mathcal{F}=\mathrm{Im}\Pi_f$ is obtained from a convolution of two real-time Green's functions of the f-fermions. Using the scaling (SYK) form of the f-fermions, one can write

$$\mathcal{F}\left(\epsilon\right)=\frac{1}{U_f}\overline{\mathcal{F}}\left(\overline{\epsilon}\right), \tag{20}$$

where $\overline{\mathcal{F}}$ is a dimensionless function of the dimensionless variable $\overline{\epsilon} = \frac{\epsilon}{T}$. Then, by rescaling $\epsilon_1, ..., \epsilon_5, \omega_n, \omega_m$ and Ω_l by T, we see that

$$\delta\Pi_J^x\left(i\overline{\Omega}_l\right) \sim \eta \frac{U_{cf}^2}{U_f W_c^2} \nu_0^2 v_F^4 \int_{\overline{\epsilon}_1,\overline{\epsilon}_2,\overline{\epsilon}_3,\overline{\epsilon}_4,\overline{\epsilon}_5} \mathcal{S}_c\left(\epsilon_1,\epsilon_2\right) \overline{\mathcal{F}}\left(\overline{\epsilon}_3\right) \mathcal{S}_c\left(\epsilon_4,\epsilon_5\right). \tag{21}$$

$$\times T^2 \sum_{n,m\in\mathbb{Z}} \frac{1}{\overline{\epsilon}_1 - i\overline{\Omega}_l - i\overline{\omega}_n} \frac{1}{\overline{\epsilon}_2 - i\overline{\omega}_n} \frac{1}{\overline{\epsilon}_3 - i\overline{\omega}_n + i\overline{\omega}_m} \frac{1}{\overline{\epsilon}_4 - i\overline{\Omega}_l - i\overline{\omega}_m} \frac{1}{\overline{\epsilon}_5 - i\overline{\omega}_m}. \tag{22}$$

Here the overline denotes the rescaled variables.

To obtain the scaling behavior of $\delta\sigma$ in the limit $T \gg \Gamma$, we set $\Gamma = 0$, such that the self-energy has the scaling MFL form, $\Sigma_R\left(\epsilon_i\right) \approx T\overline{\Sigma}_{cf,R}\left(\overline{\epsilon}_i\right)$, which means that

$$\mathcal{S}_c\left(\epsilon_i,\epsilon_j\right) = \frac{1}{T}\overline{\mathcal{S}}_c\left(\overline{\epsilon}_i,\overline{\epsilon}_j\right). \tag{23}$$

As before, the overline denotes dimensionless functions and variables. Inserting this back into the current correlator, we obtain that

$$\delta\Pi_J^x\left(i\overline{\Omega}_l\right) \sim \eta \frac{U_{cf}^2}{U_f W_c^2 g^4} \nu_0^2 v_F^4 \int_{\overline{\epsilon}_1,\overline{\epsilon}_2,\overline{\epsilon}_3,\overline{\epsilon}_4,\overline{\epsilon}_5} \overline{\mathcal{S}}_c\left(\overline{\epsilon}_1,\overline{\epsilon}_2\right) \overline{\mathcal{F}}\left(\overline{\epsilon}_3\right) \overline{\mathcal{S}}_c\left(\overline{\epsilon}_4,\overline{\epsilon}_5\right). \tag{24}$$

$$\times \sum_{n,m\in\mathbb{Z}} \frac{1}{\overline{\epsilon}_1 - i\overline{\Omega}_l - i\overline{\omega}_n} \frac{1}{\overline{\epsilon}_2 - i\overline{\omega}_n} \frac{1}{\overline{\epsilon}_3 - i\overline{\omega}_n + i\overline{\omega}_m} \frac{1}{\overline{\epsilon}_4 - i\overline{\Omega}_l - i\overline{\omega}_m} \frac{1}{\overline{\epsilon}_5 - i\overline{\omega}_m}. \tag{25}$$

We can now observe that $\delta\Pi_J^x\left(i\overline{\Omega}_l\right)$ is independent of T. Using Eq. (12), it follows that

$$\delta\sigma \sim \eta \frac{U_f v_F^4}{W_c^2 U_{cf}^2 \nu_0^2} \times \frac{1}{T} \times (\text{dimensionless integral}). \tag{26}$$

Hence the correction $\delta\sigma$ scales as $1/T$, similarly to σ. For the thermal conductivity, the derivation is similar, and gives $\delta\kappa \sim$ const when $T \gg \Gamma$. The saturation value of the Lorenz ratio is thus altered by vertex corrections.

QUANTUM BOLTZMANN EQUATION

Here we outline the idea behind the QBE for the generalized Fermi distribution function, following similar steps to Refs. [5, 6].

Derivation of QBE

We begin by introducing the non-equilibrium Green's functions $\tilde{G}$ and $\tilde{\Sigma}$ that satisfy the Dyson's equation:

$$\tilde{G} = \tilde{G}_0 + \tilde{G}_0 \tilde{\Sigma} \tilde{G} \tag{27}$$

where

$$\tilde{G} = \begin{pmatrix} G_t & -G^< \\ G^> & -G_{\bar{t}} \end{pmatrix} \tag{28}$$

and similarly for $\tilde{\Sigma}$. G_0 denotes the non-interacting Green's function. As in [6], the multiplication in Eq. (27) denotes integration over a shared space-time variable. In addition,

$$G^>(1,2) = -i\left\langle c(1)\, c^\dagger(2)\right\rangle; \tag{29}$$

$$G^<(1,2) = i\left\langle c^\dagger(2)\, c(1)\right\rangle; \tag{30}$$

$$G_t(1,2) = \Theta(t_1 - t_2)\, G^>(1,2) + \Theta(t_2 - t_1)\, G^<(1,2); \tag{31}$$

$$G_{\bar{t}}(1,2) = \Theta(t_2 - t_1)\, G^>(1,2) + \Theta(t_1 - t_2)\, G^<(1,2), \tag{32}$$

where $1 = (\boldsymbol{r}_1, t_1)$ and similarly for 2. Note that

$$G^R = G_t - G^< = G^> - G_{\bar{t}}; \tag{33}$$
$$G^A = G_t - G^> = G^< - G_{\bar{t}}, \tag{34}$$

where $G^{R(A)}$ is the familiar retarded (advanced) Green's functions. The derivation proceeds by changing to center-of-mass and relative coordinates:

$$\left(\boldsymbol{R}, \bar{t}\right) = \frac{1}{2}\left(1+2\right), \quad \left(\boldsymbol{r}, t\right) = 1 - 2. \tag{35}$$

To derive a self-consistent Dyson's equation for $G^<$, we consider the Fourier transform with respect to the relative coordinates:

$$\tilde{G}\left(\boldsymbol{k}, \omega, \boldsymbol{R}, \bar{t}\right) = \int_{\boldsymbol{r}} e^{i\boldsymbol{k}\boldsymbol{r}} \int_t e^{i\omega t} \tilde{G}\left(\boldsymbol{r}, t, \boldsymbol{R}, \bar{t}\right). \tag{36}$$

In the following we slightly abuse the notation above by relabelling the center-of-mass coordinates by $\boldsymbol{r}$ and t. In addition, we will occasionally ignore these arguments for brevity. Note that in thermal equilibrium,

$$G^<\left(\boldsymbol{k}, \omega\right) = i f_0\left(\omega\right) \mathcal{A}\left(\boldsymbol{k}, \omega\right) \tag{37}$$
$$G^>\left(\boldsymbol{k}, \omega\right) = -i\left(1 - f_0\left(\omega\right)\right) \mathcal{A}\left(\boldsymbol{k}, \omega\right) \tag{38}$$

where f_0 is the equilibrium Fermi distribution at some temperature, and the spectral function $\mathcal{A}\left(\boldsymbol{k}, \omega\right) = -i\left(G^R\left(\boldsymbol{k}, \omega\right) - G^A\left(\boldsymbol{k}, \omega\right)\right.$

Following [5, 6], the Dyson equation for $G^<$ is given by

$$\left[\omega - \epsilon_{\boldsymbol{k}} - \mathrm{Re}\Sigma^R, G^<\right] - \left[\Sigma^<, \mathrm{Re}G^R\right] = \Sigma^> G^< - G^> \Sigma^<, \tag{39}$$

where the multiplication here is standard, e.g., $\Sigma^> G^< = \Sigma^>\left(\boldsymbol{k}, \omega, \boldsymbol{r}, t\right) G^<\left(\boldsymbol{k}, \omega, \boldsymbol{r}, t\right)$, and we have introduced the generalized Poisson brackets,

$$[A, B] = \partial_\omega A \partial_t B - \partial_t A \partial_\omega B + \nabla_{\boldsymbol{r}} A \cdot \nabla_{\boldsymbol{k}} B - \nabla_{\boldsymbol{k}} A \cdot \nabla_{\boldsymbol{r}} B. \tag{40}$$

To derive the QBE, we use the fact that Σ_R is momentum independent in the low-energy limit and note that the spectral function of c-fermions,

$$\mathcal{A}_c\left(\omega, \boldsymbol{k}\right) = \frac{-2\mathrm{Im}\Sigma_R\left(\omega\right)}{\left(\omega - \varepsilon_{\boldsymbol{k}} - \mathrm{Re}\Sigma_R\left(\omega\right)\right)^2 + \left(\mathrm{Im}\Sigma_R\left(\omega\right)\right)^2}, \tag{41}$$

is sharply peaked as a function of $\varepsilon_{\boldsymbol{k}}$ at the FS for sufficiently small ω. In addition, we note that $\int \frac{d\varepsilon}{2\pi}\mathcal{A}_c = 1$. Assuming that these features persist if the system is sufficiently close to local equilibrium (limiting ourselves to linear response), it follows that by changing the integration variables from $\boldsymbol{k}$ to $\varepsilon \equiv \varepsilon_{\boldsymbol{k}}$ and $\hat{\boldsymbol{k}}$ we can define a generalized distribution function,

$$f_c\left(\hat{\boldsymbol{k}}, \omega, \boldsymbol{r}, t\right) \equiv -i \int \frac{d\varepsilon}{2\pi} G_c^<\left(\hat{\boldsymbol{k}}, \varepsilon, \omega, \boldsymbol{r}, t\right), \tag{42}$$

that describes the distribution of c-fermions with energy ω at position $\boldsymbol{r}$ and time t. Similarly, $1 - f_c\left(\hat{\boldsymbol{k}}, \omega, \boldsymbol{r}, t\right) = i\int \frac{d\varepsilon}{2\pi} G_c^>\left(\varepsilon, \omega, \boldsymbol{r}, t\right)$.

By defining the generalized distribution function (42) together with the above assumptions we effectively restrict the momentum of the c-fermions to the FS, an approximation known as the Prange-Kadanoff (PK) reduction scheme. The PK reduction can be consistently applied provided that the spectral function is sharply peaked at the FS as a function of ε. More precisely, assuming that the typical momentum transfer due to different scattering mechanisms can be characterized by a ball of radius $q_*(T)$, the PK reduction is consistent if $|\mathrm{Im}\Sigma_R\left(\omega \lesssim T\right)| \ll v_F q_*(T)$ for all scattering mechanisms. In our case, the consistency of the PK reduction at low T follows from the locality of the f-fermions, namely, $q_* = k_F$ and indeed in the T-window of interest we have that $\Gamma, T \ll v_F k_F \sim E_F$. More generally, we can see that in the presence of disorder scattering, the PK reduction is consistent if the momentum dependence of the single-particle scattering rate is sufficiently weak.

To obtain the QBE for f_c, we consider the equation of motion for $G^<$. Restricting ourselves to slowly varying force fields, the QBE is obtained from a gradient expansion [6] followed by an integration over ε of Eq. (39) [5], from which we arrive at

$$\mathcal{D} f_c\left(\hat{\boldsymbol{k}}, \omega, \boldsymbol{r}, t\right)=\mathcal{I}_{\text{coll}}\left[f_c\left(\hat{\boldsymbol{k}}, \omega, \boldsymbol{r}, t\right)\right], \tag{43}$$

where we have introduced the differential operator

$$\mathcal{D} \equiv\left(1-\partial_\omega \operatorname{Re} \Sigma_R\right) \partial_t+\partial_t \operatorname{Re} \Sigma_R \partial_\omega-\nabla_{\boldsymbol{r}} \operatorname{Re} \Sigma_R \cdot \nabla_{\boldsymbol{k}_F}+\nabla_{\boldsymbol{k}_F}\left(\varepsilon_{\boldsymbol{k}}+\operatorname{Re} \Sigma_R\right) \cdot \nabla_{\boldsymbol{r}},$$

and $\nabla_{\boldsymbol{k}_F} g \equiv \nabla_{\boldsymbol{k}} g|_{\boldsymbol{k} \in \mathrm{FS}}$ for some function g. The collision integral is given by $\mathcal{I}_{\text{coll}}=\int_{\varepsilon_{\boldsymbol{k}}} \Sigma^{>} G^{<}-G^{>} \Sigma^{<}$. Note that $\mathcal{I}_{\text{coll}}=\mathcal{I}_{\text{dis}}+\mathcal{I}_{cf}$, where $\mathcal{I}_{\text{dis}}$ and $\mathcal{I}_{cf}$ denotes the contributions due to disorder and the cf-interactions, respectively. Explicitly, the self-energies are given by

$$\Sigma_{\text{dis}}^{<(>)}(\boldsymbol{k}, \omega, \boldsymbol{r}, t) =\int_{\boldsymbol{q}} W_{\boldsymbol{k}-\boldsymbol{q}}^2 G^{<(>)}(\boldsymbol{q}, \omega, \boldsymbol{r}, t), \tag{44}$$

and

$$\Sigma_{cf}^{<}(\boldsymbol{k}, \omega, \boldsymbol{r}, t) = U_{cf}^2 \int_{\boldsymbol{q}, \nu} \widetilde{\Upsilon}(\boldsymbol{q}) \operatorname{Im} \Pi_f^R(\nu)\left\{\left(n_0(\nu)+1\right) G^{<}(\boldsymbol{k}+\boldsymbol{q}, \omega+\nu)+n_0(\nu) G^{<}(\boldsymbol{k}+\boldsymbol{q}, \omega-\nu)\right\} \tag{45}$$

$$\Sigma_{cf}^{>}(\boldsymbol{k}, \omega, \boldsymbol{r}, t) = U_{cf}^2 \int_{\boldsymbol{q}, \nu} \widetilde{\Upsilon}(\boldsymbol{q}) \operatorname{Im} \Pi_f^R(\nu)\left\{n_0(\nu) G^{>}(\boldsymbol{k}+\boldsymbol{q}, \omega+\nu)+\left(n_0(\nu)+1\right) G^{>}(\boldsymbol{k}+\boldsymbol{q}, \omega-\nu)\right\} \tag{46}$$

Here, n_0 is the equilibrium Bose distribution function. We allow spatially correlated disorder. In addition, we allow for a spatially extended cf-interaction which introduces a momentum dependence to the scattering amplitude from the f-fermions, $\widetilde{\Upsilon}(\boldsymbol{q})$ (as in the Vertex correction section above). We discuss the validity of the PK reduction in more general terms below. For our current discussion, $\widetilde{\Upsilon}=1$ and $W_{\boldsymbol{k}-\boldsymbol{q}}^2=W^2$ which is independent of momentum. These general forms of the different parts of the self-energies will be useful later on. The collision integrals are obtained from the above via an integration over $\varepsilon_{\boldsymbol{k}}$, for example,

$$\begin{aligned}
\mathcal{I}_{cf} = {} & \nu_0 U_{cf}^2 \int_{\hat{\boldsymbol{k}}', \omega', \boldsymbol{q}, \nu} \tilde{\Upsilon}(\boldsymbol{q}) \operatorname{Im} \Pi_f^R(\nu) \times \delta\left(k_F \hat{\boldsymbol{k}}'-k_F \hat{\boldsymbol{k}}-\boldsymbol{q}\right) \\
& \times\left(\delta\left(\omega'-\omega-\nu\right)\left\{n_0(\nu)\left[1-f\left(\hat{\boldsymbol{k}}', \omega'\right)\right] f\left(\hat{\boldsymbol{k}}, \omega\right)-\left[1+n_0(\nu)\right] f\left(\hat{\boldsymbol{k}}', \omega'\right)\left[1-f\left(\hat{\boldsymbol{k}}, \omega\right)\right]\right\}\right. \\
& \left.+\ \delta\left(\omega'-\omega+\nu\right)\left\{\left[1+n_0(\nu)\right]\left[1-f\left(\hat{\boldsymbol{k}}', \omega'\right)\right] f\left(\hat{\boldsymbol{k}}, \omega\right)-n_0(\nu) f\left(\hat{\boldsymbol{k}}', \omega'\right)\left[1-f\left(\hat{\boldsymbol{k}}, \omega\right)\right]\right\}\right).
\end{aligned} \tag{47}$$

At this point we can explicitly see that the momentum of the c-fermions is restricted to the Fermi surface: $\mathcal{I}_{cf}=\mathcal{I}_{cf}\left(\hat{\boldsymbol{k}}, \omega, \boldsymbol{r}, t\right)$ (and similarly for $\mathcal{I}_{\text{dis}}$).

It is worthwhile to comment on two subtle points in the above derivation: (i) The elimination of the second term on the LHS of Eq. (39) (i.e. $\left[\Sigma^{<}, \operatorname{Re} G^R\right]$) is due to the fact that we have assumed a particle-hole symmetric form for the density of states (i.e., a constant DOS $\nu(\varepsilon) \approx \nu_0$, extending from $-W_c/2$ to $W_c/2$, where W_c is the itinerant electron bandwidth). For a more generic DOS, the corresponding correction to this approximation is of the order of $|\Sigma^{<}|/W_c$. The QBE is valid when this correction is small, namely, when the scattering rate is small compared to the Fermi energy, similarly to the standard Boltzmann equation. (ii) Note that in order to integrate over $\varepsilon_{\boldsymbol{k}}$ in the collision integral, we have used the fact that the scattering rate depends weakly on $\varepsilon_{\boldsymbol{k}}$ at low energies. This is consistent with the fact that the internal frequency, ν, is always restricted to be of the order of T, given that the external frequency $\omega \sim T$ and the system is close to thermal equilibrium.

Variational formulation

A direct solution of this QBE is clearly a non-trivial task. Instead, we will compute the resitivities via a variational formulation of the QBE. The validity of the WF law at $T \to 0$ essentially follows from the fact that the elastic scattering term, $\mathcal{I}_{\text{dis}}$, dominates the inelastic term $\mathcal{I}_{cf}$, similarly to the conventional QBE description of FLs. To demonstrate this, we linearize the QBE in a manner that allows us to utilize a variational formulation of the QBE

along the lines of [2, 5]. We parameterize the deviation from equilibrium with the function $\phi\left(\hat{\boldsymbol{k}},\omega,\boldsymbol{r},t\right)$, such that the full distribution function is approximated as follows,

$$f_c\left(\hat{\boldsymbol{k}},\omega,\boldsymbol{r},t\right)=f_0\left(\omega,\boldsymbol{r},t\right)-\phi\left(\hat{\boldsymbol{k}},\omega,\boldsymbol{r},t\right)\partial_\omega f_0\left(\omega,\boldsymbol{r},t\right). \tag{48}$$

The local equilibrium distribution f_0 nullifies the collision integrals by definition. Also note that f_0 could depend on space and time via local temperature $1/\beta\left(\boldsymbol{r},t\right)$ or chemical potential $\mu\left(\boldsymbol{r},t\right)$ [5]. Using the form (48), and following the steps in Ref. [5], one can show that the linearized QBE in the presence of a uniform electric field $\boldsymbol{E}$, which we will consider for the computation of the electrical resistivity, is given by

$$-\boldsymbol{E}\cdot\hat{\boldsymbol{k}}v_F\partial_\omega f_0=\mathcal{I}_{\text{coll}}. \tag{49}$$

And similarly for an applied uniform thermal gradient, the linearized QBE we will consider for the computation of the thermal resistivity is given by

$$\nabla_{\boldsymbol{k}_F}\varepsilon_{\boldsymbol{k}}\cdot\nabla_{\boldsymbol{r}}f_0=\mathcal{I}_{\text{coll}}. \tag{50}$$

Remarkably, the form (48) enables us to relate the thermal and electrical resistivities to a variational problem in the function ϕ. Specifically, the physical values of ρ and ρ_{th} correspond to ϕ that minimizes the functionals ($a=\text{el},\text{th}$) $\mathcal{F}^a[\phi]=\mathcal{F}^a_{\text{dis}}[\phi]+\mathcal{F}^a_{cf}[\phi]$, where the disorder and cf-interaction contributions to the resistivities are denoted by $\mathcal{F}_{\text{dis}}$ and $\mathcal{F}_{cf}$, respectively. The derivation of the variational formulation is analogous to the one in Refs. [2, 5]. We therefore state the final expressions for the resistivities. To do so, we must introduce an inner product defined as

$$\langle g,h\rangle\equiv\nu_0\int_{\hat{\boldsymbol{k}},\omega}g\left(\hat{\boldsymbol{k}},\omega\right)h\left(\hat{\boldsymbol{k}},\omega\right) \tag{51}$$

for some functions g and h. In addition we define the operators $\mathcal{P}_a$ as

$$\mathcal{P}_a\phi\equiv\nu_0\int_{\hat{\boldsymbol{k}}',\omega'}\left(\phi\left(\hat{\boldsymbol{k}},\omega\right)-\phi\left(\hat{\boldsymbol{k}}',\omega'\right)\right)P_a\left(\hat{\boldsymbol{k}},\omega,\hat{\boldsymbol{k}}',\omega'\right) \tag{52}$$

with P_a being the equilibrium transitional rates related to the different scattering mechanisms. Using these definitions, the electrical resistivities are conveniently given by

$$\mathcal{F}^a\left[\phi\right]=\mathcal{F}^a_{\text{dis}}\left[\phi\right]+\mathcal{F}^a_{cf}\left[\phi\right]=\frac{\langle\phi,\mathcal{P}_{\text{dis}}\phi\rangle}{\left|\langle\phi,X^a\rangle\right|^2}+\frac{\langle\phi,\mathcal{P}_{cf}\phi\rangle}{\left|\langle\phi,X^a\rangle\right|^2},\quad a=\text{el},\text{th}. \tag{53}$$

Here,

$$\langle\phi,\mathcal{P}_{\text{dis}}\phi\rangle=\nu_0\beta\int_{\omega,\hat{\boldsymbol{k}},\omega',\hat{\boldsymbol{k}}'}W^2\left(\phi\left(\hat{\boldsymbol{k}},\omega\right)-\phi\left(\hat{\boldsymbol{k}}',\omega'\right)\right)^2\delta\left(\omega-\omega'\right)f_0\left(\omega\right)\left(1-f_0\left(\omega'\right)\right); \tag{54}$$

$$\begin{aligned}\langle\phi,\mathcal{P}_{cf}\phi\rangle &= 2U_{cf}^2\nu_0\beta\int_{\omega,\hat{\boldsymbol{k}},\omega',\hat{\boldsymbol{k}}',\nu,\boldsymbol{q}}\tilde{\Upsilon}\left(\boldsymbol{q}\right)\text{Im}\Pi_f^R\left(\nu\right)f_0\left(\omega\right)\left(1-f_0\left(\omega'\right)\right)n_0\left(\nu\right)\\ &\times\left(\phi\left(\hat{\boldsymbol{k}},\omega\right)-\phi\left(\hat{\boldsymbol{k}}',\omega'\right)\right)^2\delta\left(k_F\hat{\boldsymbol{k}}'-k_F\hat{\boldsymbol{k}}-\boldsymbol{q}\right)\delta\left(\omega'-\omega-\nu\right),\end{aligned} \tag{55}$$

where the factor of 2 comes from the equal contribution of an emission and absorption of a 'Π_f-boson' [5], and

$$\left|\langle\phi,X^{\text{el}}\rangle\right|^2=\left|\nu_0\int_{\hat{\boldsymbol{k}},\omega}v_F\hat{\boldsymbol{k}}\phi\left(\hat{\boldsymbol{k}},\omega\right)\partial_\omega f_0\left(\omega\right)\right|^2; \tag{56}$$

$$\left|\langle\phi,X^{\text{th}}\rangle\right|^2=\left|\nu_0\int_{\hat{\boldsymbol{k}},\omega}v_F\hat{\boldsymbol{k}}\phi\left(\hat{\boldsymbol{k}},\omega\right)\omega\partial_\omega f_0\left(\omega\right)\right|^2. \tag{57}$$

Anticipating the dominance of elastic scattering as $T\to0$, we may use the variational ansatzes:

$$\phi^{\text{el}}_{\text{dis}}\left(\hat{\boldsymbol{k}},\omega\right)=\eta\boldsymbol{u}\cdot\hat{\boldsymbol{k}},\quad\phi^{\text{th}}_{\text{dis}}\left(\hat{\boldsymbol{k}},\omega\right)=\eta\omega\boldsymbol{v}\cdot\hat{\boldsymbol{k}}, \tag{58}$$

where $\boldsymbol{u}$ ($\boldsymbol{v}$) denotes a unit vector in the direction of the electrical (heat) current and η is a small parameter. By inserting the ansatzes (58) into Eq. (53) with the above definitions, we can explicitly see that the validity of the WF law at $T \to 0$ follows from the dominance of the T-independent elastic scattering contribution, $\mathcal{F}_{\rm dis}$, over the inelastic term, $\mathcal{F}_{cf}$. In addition, a simple power counting (as in Eq. 20) shows that the scaling form of Π_f leads to T–linear resitivities. In total, we confirm that

$$\rho \approx \mathcal{F}^{\rm el}_{\rm dis}[\phi^{\rm el}_{\rm dis}] + \mathcal{F}^{\rm el}_{cf}[\phi^{\rm el}_{\rm dis}] = \rho_0 + AT, \tag{59}$$

and similarly $\rho_{\rm th} = \rho_{\rm th,0} + BT$. Importantly, Eq. (59) captures the physical leading low-T behavior, rather than serving as an upper bound. Indeed, consider leading correction to $\phi^{\rm el}_{\rm dis}$, $\delta\phi^{\rm el}$, such that the minimizer of the resistivity functional is $\phi^{\rm el} = \phi^{\rm el}_{\rm dis} + \delta\phi^{\rm el}$. Then, by expanding the right-hand-side of Eq. (59) in $\delta\phi^{\rm el}$ and using the fact that $\phi^{\rm el}_{\rm dis}$ minimizes $\mathcal{F}^{\rm el}_{\rm dis}[\phi^{\rm el}]$, we obtain that the contribution due to $\delta\phi^{\rm el}$ is subleading. In practice, $\phi_{\rm dis}$ is expected to minimizes the full functional $\mathcal{F}$ in the case of a local cf-interaction.

So far, the QBE provided a simple perspective for the validity of the WF law in the case of local cf-interactions, i.e., $\Upsilon_{\boldsymbol{r},\boldsymbol{r}'} = \delta_{\boldsymbol{r},\boldsymbol{r}'}$ (such that $\widetilde{\Upsilon} = 1$), for which Π_f is completely uniform in momentum space. But in fact, since the key ingredient in our derivation was the PK reduction scheme, the above discussion can be generalized to a class of deformed models with spatially extended cf-interactions. See the main text (MFL section) for an example. Indeed, under such deformations, the scattering off of f-fermions obtains a momentum dependence, $\Pi_f(\nu) \to \Pi_f(\boldsymbol{q},\nu) \equiv \widetilde{\Upsilon}(\boldsymbol{q})\,\Pi_f(\nu)$. However, as long as this momentum dependence is not singular, namely, it can be written as $\widetilde{\Upsilon}(\boldsymbol{q}) \sim 1 + \eta h(\boldsymbol{q})$ with a sufficiently small η and smooth h, the low-energy MFL form of the self-energy of the c-fermions does not change (as demonstrated above). And most importantly, since the PK reduction scheme is valid, we can repeat the analysis above. It thus follows that this class of deformations, and in particular the example presented in the main text (MFL section), also obeys Eq. (5) in the main text.

For completeness, let us note that the main effect of spatially extended cf-interactions is to change the nonuniversal prefactor of the leading $-T$ term in $L(T) - L_0$. That is, it modifies the coefficients A and B of the resistivities above. For the electrical resistivity, this is due to the suppressed contribution of small angle scattering events. This can be seen explicitly by noticing that, in this case, ϕ is frequency independent [see Eq. (58)] and the momentum and frequency integrations in Eq. (55) factorize such that

$$\langle \phi, \mathcal{P}_{cf}\phi\rangle = \int_{\hat{\boldsymbol{k}},\hat{\boldsymbol{k}}',\boldsymbol{q}} \tilde{\Upsilon}(\boldsymbol{q}) \left(\phi\left(\hat{\boldsymbol{k}}\right) - \phi\left(\hat{\boldsymbol{k}}'\right)\right)^2 \delta\left(k_F\hat{\boldsymbol{k}}' - k_F\hat{\boldsymbol{k}} - \boldsymbol{q}\right) \times (\text{frequency integrals}). \tag{60}$$

For $\Upsilon = 1$ we retrieve the familiar $1 - \cos\theta_{\boldsymbol{k},\boldsymbol{k}'}$ weighting factor, while any momentum dependence in Υ will change the weighting and hence the overall prefactor. The T-scaling is determined by the frequency intergrals and is hence unaffected. Similar, yet more involved, consideration can be applied to the thermal resistivity [2].

The validity of PK reduction was the key ingredient to the derivation above, namely, the same analysis can be applied to generic weakly disordered MFLs (and NFLs), provided that the momentum-dependence of the inelastic scattering rate is sufficiently weak (which enables the PK reduction). Furthermore, this means that the low-T deviation from the WF law (Eq. (5) in the main text) is not a fine-tuned feature of our model, and could serve as a generic criterion for strangeness, as claimed in the main text.

Lastly, notice that by spatially extending that range of Υ we reduce the characteristic momentum transfer to a narrower region in the Brillouin zone. Denoting the radius of this region by q_*, the validity condition for the PK reduction can be roughly estimated as $v_F q_* \gg \Gamma$. It is interesting that by further extending the range of Υ, such that $v_F q_* \sim \Gamma$, we effectively invalidate the PK reduction scheme. The PK reduction scheme cannot be consistently used in this case. This scenario is reminiscent of the MFL model studied in [7].

Generalization to the transverse Lorenz ratio

We obtain the transverse conductivities by a solving the QBE in the presence of a magnetic field for a local $cf-$interaction (introducing extended $cf-$interaction does not change the qualitative physical picture). The reason for this alternative derivation is that the variational principal is not directly valid in the presence of a magnetic field since the operators P_a are not self-adjoint [2]. Physically, this is related to the fact that time-reversal symmetry is broken such that the probability of a scattering of an excitation $(\hat{\boldsymbol{k}},\omega)$ to an excitation $(\hat{\boldsymbol{k}'},\omega')$ is not equal to the probability in the reverse direction. Nevertheless, the direct solution of the QBE is shows that the transverse Lorenz ratio follows the same $T-$scaling is the longitudinal one provided that the PK reduction holds.

The introduction of a magnetic field follows the same steps as in the case of an electric field. Specifically, we introduce an electromagnetic vector potential via minimal coupling, $\varepsilon_{\boldsymbol{k}} \to \varepsilon_{\boldsymbol{k}+\boldsymbol{A}}$ where $\boldsymbol{A} = \boldsymbol{A}_1 + \boldsymbol{A}_2$ such that $-d\boldsymbol{A}_1/dt = \boldsymbol{E}$ and $\nabla \times \boldsymbol{A}_2 = \boldsymbol{B}$. Then, the QBE is obtained in a similar fashion to the derivation above, where the introduction of electromangetic fields is done via a change of the COM coordinates $\boldsymbol{k} \mapsto \boldsymbol{k} + \boldsymbol{A}$; see e.g. [8]. The QBE then takes the form

$$\widetilde{\mathcal{D}} f_c\left(\hat{\boldsymbol{k}}, \omega, \boldsymbol{r}, t\right) = \mathcal{I}_{\text{coll}}\left[f_c\left(\hat{\boldsymbol{k}}, \omega, \boldsymbol{r}, t\right)\right] \tag{61}$$

where $\widetilde{\mathcal{D}} = \mathcal{D} + \nabla_{\boldsymbol{k}_F}\varepsilon_{\boldsymbol{k}} \cdot \boldsymbol{E}\partial_\omega + (\nabla_{\boldsymbol{k}_F}\varepsilon_{\boldsymbol{k}} \times \boldsymbol{B}) \cdot \nabla_{\boldsymbol{k}_F}$. Similarly to the conventional Boltzmann equation, the magnetic part of the Lorentz force nullifies the equilibrium distribution and acts nontrivially only on the non-equilibrium piece.

To proceed, we insert the parametrization of the full distribution, $f_c \equiv f_0 + \delta f$, into $\mathcal{I}_{\text{coll}}$. Then, we use (a) the fact that by definition $\mathcal{I}_{\text{coll}}\left[f_0\right] = 0$ [9], and (b) the fact that $\delta f(-\hat{\boldsymbol{k}}) = -\delta f(\hat{\boldsymbol{k}})$, which implies that the terms proportional to $\delta f(\hat{\boldsymbol{k}}^{'})$ vanish in the integration over $\hat{\boldsymbol{k}}^{'}$. It is then straightforward to express the collision integral in terms of the self-energy of the $c-$fermions:

$$\mathcal{I}_{\text{coll}}[f] = 2\Sigma_R''(\omega)\delta f. \tag{62}$$

Finally the QBE for in the presence of an electric and magnetic field is given by

$$v_F\hat{\boldsymbol{k}} \cdot \boldsymbol{E}\partial_\omega f_0\left(\omega\right) + v_F\left(\hat{\boldsymbol{k}} \times \boldsymbol{B}\right) \cdot \nabla_{\boldsymbol{k}_F}\delta f\left(\hat{\boldsymbol{k}}, \omega\right) = 2\delta f\left(\hat{\boldsymbol{k}}, \omega\right)\Sigma_R^{''}\left(\omega\right). \tag{63}$$

The QBE in the presence of a small thermal gradient is identical to the above with the replacement $\boldsymbol{E} \to \beta\omega\nabla_{\boldsymbol{r}}T$. The solution of the QBE is obtained by inserting the ansatz [2] $\delta f\left(\hat{\boldsymbol{k}}, \omega\right) = k_F\hat{\boldsymbol{k}} \cdot \boldsymbol{\delta f}\left(\omega\right)$, which yields (in agreement with [10])

$$\delta f_i\left(\omega\right) = \frac{v_F}{k_F}\partial_\omega f_0\left(\omega\right)\left(2\Sigma_R''\left(\omega\right)\delta_{ij} + \epsilon_{ij}B\frac{v_F}{k_F}\right)^{-1}F_j \tag{64}$$

with $F_j = E_j, \beta\omega\partial_j T$ for an applied electric field and thermal gradient, respectively (the magnetic field is assumed to point along $\hat{z}$, i.e. out of the plane), and ϵ_{ij} is the antisymmetric tensor in two dimensions. We recall that, in our model, by definition the (expectation values of the) electrical and thermal currents (per flavor) are given by

$$J_{\text{el},i} = -\nu_0\int_{\hat{\boldsymbol{k}},\omega}\boldsymbol{v}_F\delta f\left(\hat{\boldsymbol{k}}, \omega\right) \tag{65}$$

and

$$J_{\text{th},i} = -\nu_0\int_{\hat{\boldsymbol{k}},\omega}\boldsymbol{v}_F\omega\delta f\left(\hat{\boldsymbol{k}}, \omega\right). \tag{66}$$

We can therefore obtain the transverse electrical and thermal conductivities,

$$\sigma_{xy} = \frac{v_F^2\nu_0}{16T}\int\frac{d\epsilon}{2\pi}\text{sech}^2\left(\frac{\epsilon}{2T}\right)\frac{\omega_c/2}{\Sigma_R''\left(\epsilon\right)^2 + \left(\omega_c/2\right)^2} \tag{67}$$

and

$$\kappa_{xy} = \frac{v_F^2\nu_0}{16T^2}\int\frac{d\epsilon}{2\pi}\text{sech}^2\left(\frac{\epsilon}{2T}\right)\epsilon^2\frac{\omega_c/2}{\Sigma_R''\left(\epsilon\right)^2 + \left(\omega_c/2\right)^2}, \tag{68}$$

where the cyclotron frequency is given by $\omega_c = \left(v_F/k_F\right)B$.

We can now explicitly see that $L_{xy} - L_0 \propto -T$ in agreement with Eq. (5) in the main text. For example, in the simplest case where the magnetic field is sufficiently small ($\omega_c \ll \Gamma$), the conductivities obey the relation $\alpha_{xy}(T) = (\omega_c/2\Gamma)\alpha_{xx}(T)$ ($\alpha = \sigma, \kappa$) to leading order in ω_c, which automatically guarantees the desired behavior. Note also that even when $\Gamma \to 0$ the WF law is obeyed (ω_c takes the role of the disorder term) but with a deviation that scales as T^2.

Analogously to the longitudinal case, the leading T-scaling of the transport rates is governed by the form of $\text{Im}\Pi_f^R(\nu)$ in $\mathcal{I}_{cf}$, which is unaffected by the introduction of extended interactions. Hence, as in the longitudinal case,

spatially extended cf-interactions changes the prefactor of the $-T$ term in $L_{xy}(T) - L_0$, but not its scaling form. More generally, the T-scaling remains unchanged as long as the inelastic scattering mechanism has sufficiently weak momentum dependence. This is exactly the validity condition of the PK reduction. We thus observe that, similarly to the discussion on the longitudinal Lorenz ratio, our conclusion holds for models of weakly disordered MFLs (of NFLs) where the PK reduction scheme can be applied. In other words, the leading deviation of the transverse Lorenz ratio satisfies the same generic behavior as the longitudinal Lorenz ratio, provided that the PK reduction is valid.

* Corresponding author: evyatar.tulipman@weizmann.ac.il

[1] A. Rosch, "Interplay of Disorder and Spin Fluctuations in the Resistivity near a Quantum Critical Point," Phys. Rev. Lett. **82**, 4280–4283 (1999).
[2] John M Ziman, "Electrons and Phonons: The Theory of Transport Phenomena in Solids (oxford university press, 1960)," Oxford University Press (1960).
[3] R. Hlubina and T. M. Rice, "Resistivity as a function of temperature for models with hot spots on the Fermi surface," Phys. Rev. B **51**, 9253–9260 (1995).
[4] Debanjan Chowdhury, Yochai Werman, Erez Berg, and T. Senthil, "Translationally Invariant Non-Fermi-Liquid Metals with Critical Fermi Surfaces: Solvable Models," Phys. Rev. X **8**, 031024 (2018).
[5] Cody P. Nave and Patrick A. Lee, "Transport properties of a spinon Fermi surface coupled to a U(1) gauge field," Phys. Rev. B **76**, 235124 (2007).
[6] Gerald D. Mahan, Many-Particle Physics, 3rd ed., Physics of Solids and Liquids (Springer US, 2000).
[7] Haoyu Guo, Ilya Esterlis, Aavishkar A. Patel, and Subir Sachdev, "Large N theory of critical Fermi surfaces II: conductivity," (2022), 10.48550/arXiv.2207.08841, Preprint at `http://arxiv.org/abs/2207.08841`.
[8] J. Rammer and H. Smith, "Quantum field-theoretical methods in transport theory of metals," Rev. Mod. Phys. **58**, 323–359 (1986).
[9] Alex Kamenev, Field Theory of Non-Equilibrium Systems (Cambridge University Press, 2011).
[10] Aavishkar A. Patel, John McGreevy, Daniel P. Arovas, and Subir Sachdev, "Magnetotransport in a Model of a Disordered Strange Metal," Phys. Rev. X **8**, 021049 (2018).
